# pH-Induced 3D Printable Chitosan Hydrogels for Soft Actuation

**DOI:** 10.3390/polym14030650

**Published:** 2022-02-08

**Authors:** Sheila Maiz-Fernández, Leyre Pérez-Álvarez, Unai Silván, José Luis Vilas-Vilela, Senentxu Lanceros-Méndez

**Affiliations:** 1 BCMaterials (Basque Center for Materials), Applications and Nanostructures, UPV/EHU Science Park, 48940 Leioa, Spain; sheila.maiz@bcmaterials.net (S.M.-F.); unai.silvan@bcmaterials.net (U.S.); joseluis.vilas@ehu.eus (J.L.V.-V.); senentxu.lanceros@bcmaterials.net (S.L.-M.); 2 Macromolecular Chemistry Group (LABQUIMAC), Department of Physical Chemistry, Faculty of Science and Technology, University of the Basque Country, UPV/EHU, Barrio Sarriena, s/n, 48940 Leioa, Spain; 3 Ikerbasque, Basque Foundation for Science, 48009 Bilbao, Spain

**Keywords:** chitosan, 3D printing, hydrogels

## Abstract

Three-dimensional (3D) printing represents a suitable technology for the development of biomimetic scaffolds for biomedical and tissue engineering applications. However, hydrogel-based inks’ printability remains a challenge due to their restricted print accuracy, mechanical properties, swelling or even cytotoxicity. Chitosan is a natural-derived polysaccharide that has arisen as a promising bioink due to its biodegradability, biocompatibility, sustainability and antibacterial properties, among others, as well as its ability to form hydrogels under the influence of a wide variety of mechanisms (thermal, ionic, pH, covalent, etc.). Its poor solubility at physiological pH, which has traditionally restricted its use, represents, on the contrary, the simplest way to induce chitosan gelation. Accordingly, herein a NaOH strong base was employed as gelling media for the direct 3D printing of chitosan structures. The obtained hydrogels were characterized in terms of morphology, chemical interactions, swelling and mechanical and rheological properties in order to evaluate the influence of the gelling solution’s ionic strength on the hydrogel characteristics. Further, the influence of printing parameters, such as extrusion speed (300, 600 and 800 mm/min) and pressure (20–35 kPa) and the cytocompatibility were also analyzed. In addition, printed gels show an electro-induced motion due to their polycationic nature, which highlights their potential as soft actuators and active scaffolds.

## 1. Introduction

Three-dimensional (3D) printing has experienced a fast development in the last decade and has become a promising manufacturing method in the biomedical and tissue engineering fields [1]. This technology represents a great advance in comparison with traditional methods, such as electrospinning or solvent casting, which typically provide uncontrolled geometries, restrict biomaterial functionality and lead to uncontrolled cell distributions, which consequently limit their use in biological applications [2]. On the contrary, 3D printing allows the production of complex and precise three-dimensional shapes and structures that are of great interest in the development of medical devices and tissue engineering scaffolds [3].

Polymeric hydrogels, which are three-dimensional structures formed by the chemical or physical crosslinking of polymeric chains [4], are able to maintain a large amount of water inside their structure without dissolving. This confers on them the ability to imitate natural tissues, and consequently, hydrogel scaffolds are considered suitable biomaterials for soft tissue regeneration [5].

Hydrogels can be based on natural or synthetic polymers; however, the use of natural polymers, such as polysaccharides, is preferred over traditional biosynthetic ones (poly-L-lactide or polyethyleneglycol, among others) due to their enhanced biological response [6], as well as their renewable nature, which is required in the scope of sustainable economy policies [7]. 

Although the 3D printing of polysaccharides has made steady progress over the past decade, it remains a challenge due to the high water content of hydrogels inks. Indeed, in order to be printable, polysaccharide inks still have to overcome their low mechanical properties and poor printing accuracy [8]. 

In this regard, one of the most important considerations in improving printability is that a fast in situ sol–gel transition must occur after the printing of the ink. Thus, before being deposited on the printing surface the bioink is presented in a liquid state, and once it is printed, as a consequence of applying an external change, this becomes a gel by an in situ-promoted physically or covalently induced crosslinking. In addition, printed soft structures must be long-lived and robust enough to self-support and accurately reproduce the designed geometry. Finally, an adequate ink must also exhibit at the same time structural integrity and controlled biodegradation at physiological conditions [9]. Consequently, research into new printing processes for polysaccharide-based inks that meet these stringent requirements is still necessary in order to make progress in this scope.

Chitosan, a biopolymer mainly obtained from chitin, which is the second-most-abundant natural polymer after cellulose, is a polysaccharide that has been studied in recent years as ink because of its biocompatibility, biodegradability, mucoadhesion, ease of functionalization and suitability for injection [10]. This polysaccharide is a weak polycation with a pKa value of 6.5 [11], and it is formed by two units (*N*-acetyl-glucosamine and glucosamine) which are randomly ordered. Since chitosan is not ionized in a pH above its pKa values, it is able to create strong inter- and intramolecular interactions, causing the precipitation of the polymer. This precipitation is related with the neutralization of the amino groups present in its chemical structure. Neutralization leads to the disappearance of the repulsive electrostatic forces that keep the polymers in the solution at acidic pH, resulting in the formation of intramolecular–intermolecular hydrogen bonds and hydrophobic interactions responsible for the precipitation of the polysaccharide [12].

Therefore, chitosan is not soluble in most solvents and conditions, including aqueous solutions or physiological pH. This fact has restricted its applicability as bioink, although some comparative studies on hydrogels’ cellular proliferation and differentiation properties have highlighted the interest of chitosan instead of alginate, which is one of the most employed polysaccharide hydrogel inks [13].

Specifically, chitosan is an advantageous polysaccharide ink because it undergoes sol–gel transitions that can be induced by different gelation processes, which confer on it versatility as a bioink [14]. In this line, chitosan can be ionotropically crosslinked with a large variety of compounds, such as citrates, and phosphates such as tripolyphosphate (TPP) are the most employed ones [15]. Complexation with other polyelectrolytes and concretely polyanions, such as alginate or hyaluronic acid, has also been demonstrated to be an efficient way to produce chitosan gelation and printable polycomplex hydrogels [16].

Indeed, the ease of functionalization of chitosan offers this polysaccharide great versatility for the development of new bioinks based on specific gelation mechanisms driven by different chitosan derivatives. Examples of this can be found in the large number of publications that report the UV crosslinking of chitosan vinyl derivatives developed for 3D printing applications [17].

Further, the poor solubility properties of chitosan at physiological conditions can also be exploited to provide gelation opportunities by means of a coagulation-mediated gelation process. As explained above, chitosan in an acidic solution rapidly precipitates at pH values above its pKa, leading to the fast formation of a hydrogel as a consequence of the neutralization of its amino groups and the promotion of hydrogen bonding and hydrophobic interactions. Nevertheless, this simple approach for chitosan hydrogel printing has been poorly addressed. Almeida et al. [18] employed 3D-printed chitosan scaffolds obtained from the gelation of a high-concentrated polymer solution by contact with a mixture of NaOH solution (8%) in ethanol (70%) to study the modulation of macrophage responses in the inflammation process. Wu et al. [19] characterized the printing process of chitosan in which in situ gelation took place by citrate action, although they observed also the positive effect of NaOH (1M) used in posterior neutralization steps on both the printability and the mechanical stability of printed complex structures [19]. Prolonged contact with NaCl solutions has been also employed as a post-printing step of chitosan bioinks in order to achieve strong mechanical properties [1]. Zhang et al. [20] also employed chitosan gelation induced by contact with a NaOH solution (3 M) and demonstrated the improvement of printed hydrogels’ mechanical properties and the restriction on post-shrinkage derived from precipitation, but in this case promoted by the addition of silk particles. Bergonzni et al. [12] investigated the combination of cryogenic printing, in which the polymer is instantaneously frozen after being extruded in a cool surface, with the gelation promoted with different agents, such as ammonia gas, a strong base KOH with variable concentrations and weak bases such as carbonate or phosphate salts. Neutralization times were determined in order to guarantee the appropriate conditions (gelation agent and concentration) to reproduce and maintain 3D-printed shapes, showing, among the solutions, that KOH and Na_2_CO_3_ lead to the lowest gelation times [12]. 

In the light of these works that demonstrate the effective printability of chitosan inks without chemical modification by contact with bases, the present work reports the room temperature direct printing of chitosan by simple contact with concentrated NaOH solutions. Besides, the effect of NaOH concentration on the printing and on the physico-chemical properties of printed structures was also evaluated. Printability, rheological, mechanical and swelling properties, the in vitro degradation profile and cytotoxicity were studied at different neutralization solution concentrations, optimizing the printing conditions that lead to an optimized performance of the printed biomaterial. On the other hand, the characteristic pH sensitivity of chitosan provides the hydrogels with the inherent ability to bend in response to an electrical stimulus, which additionally makes them suitable candidates for use as soft actuators and active scaffolds [21]. 

## 2. Materials and Methods

### 2.1. Materials

Chitosan from crab shells (Sigma-Aldrich, St. Louis, MO, USA, highly viscous, 8.7 × 10^5^ ± 4.0 × 10^4^ g/mol (PDI = 1.037), deacetylation degree of 85% determined by ^1^H-NMR) was used for the synthesis of hydrogels. The average molecular weight (M_w_) was measured by gel permeation chromatography equipped with refractive index (RID) and light-scattering (LS15 and LS90) detectors (HPLC Agilent Technologies, Agilent Technologies, Santa Clara, CA, USA). The employed column was a PolySep-GFC-P Linear 300 × 7.8 mm Phenomenex and acetic acid 0.15 M with 1 mL/min flow, and 20 μL injection volume was employed as eluent, followed a detector calibration against a poly(ethylene oxide) narrow standard (1.5 MDa). Acetic acid (for analysis, 99.8%) and lysozyme enzyme (from chicken egg white, ∼70,000 U/mg), sodium hydroxide (pure, pharma grade) and sodium phosphate monobasic (BioXtra, ≥ 99.0%) were purchased from Sigma-Aldrich, St. Louis, MO, USA. 

### 2.2. Methods

#### 2.2.1. Hydrogel Synthesis and Printability

To prepare bulk hydrogels 1.5% (*w*/*w*) solution of chitosan was prepared adding 0.75 g of chitosan to 50 mL of 0.5% (*v*/*v*) acetic acid solution with homogeneous and controlled stirring overnight. Chitosan-based bulk hydrogels were prepared by mixing 3 g of chitosan solution with NaOH solutions of different concentrations (1, 3 and 5M) under vigorous mechanical stirring. Once chitosan solution was prepared, it was loaded into the printer syringe and printed onto a Petri dish, which had previously been covered by sodium hydroxide solution (1, 3 and 5M). 

Using the CellInk HeartWare designer software, a strand of 4 cm and a square scaffold with 4 × 4 pores per side were designed (pore dimensions of 5 × 5 mm). For both strands and square scaffolds, hydrogel was printed (Cellink, INKREDIBLE+, Brighton, UK) from chitosan 1.5% (*w*/*w*) solution with a viscosity of 6092 ± 110 cP. The effect of printing speed (300, 600 and 800 mm/min), pressure (25, 30, 35 and 40 kPa) and nozzle’s diameter (22 and 25 G, corresponding to 0.41 and 0.254 mm, respectively) (Adhesive Dispensing, Ltd., Milton Keynes, UK) was evaluated on the definition of the printed structures by the determination of the uniformity factor (U), expansion ratio (α) and, in the case of the square scaffolds, the size accuracy and squareness parameters.

Uniformity factor (Equation (1)) is defined as the difference between the length of the printed structure (l) and the theoretical length (L):

Uniformity factor (U) = l/L(1)

Further, the expansion ratio (Equation (2)) indicates the ability of the bioink to spread over the printed surface. It is defined as the relation between the diameter of the printed filament (d) and the theoretical diameter of the nozzle (D):

Expansion ratio (α) = d/D (2)

The size accuracy (Equation (3)) quantifies the printing fidelity by comparing the theoretical area of the pores of the designed structure with the real area of the printed squares. This parameter is calculated taking into consideration the definition given by Di Giuseppe et al. [22]:

Size accuracy = 1 − (A_t_ − A)/A_t_ (3)

where A_t_ is the theoretical pore area (25 mm^2^), and A is the pore area of each printed square scaffold. 

On the other hand, the squareness of printed scaffolds was determined by the equation defined by Ouyang et al. [23] (Equation (4)):

Squareness = L^2^/16A (4)

where L is the perimeter of the pore, and A is the area of the printed square scaffolds.

Each structure was printed in triplicate under the same conditions, and the estimation of the different parameters was determined by the analysis of the images of the printed structures using Fiji software.

##### Gelation Time

Gelation time of hydrogels is a key factor for the materials used as bioinks since this parameter might compromise printing fidelity. It was determined following the so-called inverted tube test [24]. In short, gelation time is determined as the moment when the solution stops flowing after inverting the tube. Gelation times of the hydrogels were determined at different NaOH concentrations (1, 3 and 5 M).

#### 2.2.2. Physico-Chemical Characterization

##### Fourier-Transform Infrared Spectroscopy (FTIR)

Nicolet Nexus FTIR spectrometer (Thermo Scientific, Loughborough, UK) was employed to corroborate the formation of chitosan-based hydrogels. For this, previously dried hydrogels were analyzed by ATR, while in the case of pristine chitosan the analysis was carried out by KBr pellets. In both cases, the experiments were carried out at a resolution of 4 cm^−1^ and 32 scans per spectrum.

##### Morphological Characterization

Morphology and pore size of chitosan-based hydrogels were analyzed by scanning electron microscope (Hitachi S-4800, 150 s, 20 mA, 15 kV, zoom at ×30,000, Tokyo, Japan). Chitosan hydrogels were lyophilized (−50 °C, 0.1 mBar) and coated with a thin gold layer before SEM characterization. Finally, the average pore size was determined using ImageJ software [25], and at least 35 pores were analyzed in each sample. The error of the distribution is represented as the mean +/− standard deviation.

##### In Vitro Swelling

The swelling behaviour of hydrogels was evaluated by immersing freeze-dried chitosan hydrogels (−50 °C and 0.1 mBar) in phosphate-buffered solution (PBS) (pH = 7.4) at 37 °C in order to imitate physiological media. The swelling kinetic was evaluated by measuring the absorption of water into the hydrogels gravimetrically over time, and then the swelling ratio was measured following Equation (5):

Swelling factor = (W_s_ − W_d_)/W_d_ (5)

where, W_s_ and W_d_ are the weights of the swollen and dried hydrogels, respectively.

##### Rheology

To evaluate the dynamic rheological behaviour of prepared hydrogels oscillatory rheometry was employed using, for this aim, a rheometric scientific advanced rheometric expansion system (ARES, New Castle, PA, USA) equipped with a parallel plate geometry (25 mm of diameter). The study was carried out, firstly by a shear strain sweep to determine the linear viscoelastic region of the materials. Subsequently, frequency sweep measurements were carried out from 0.1 to 500 rad/s at a fixed strain of 1% and at a gap distance of 1.5 mm at 25 °C. Both strain and frequency sweeps were measured in triplicate.

##### Compressive Stress–Strain Tests

Compression tests were carried out at room temperature using a texture analyzer instrument (Metrotec FTM-50, Lezo, Spain) equipped with a 20 N load cell. Measurements were carried out under progressive compression at a rate of 1 mm/min until breaking. Compression moduli were calculated from the slope of the linear portion (40–60% strain range) of the stress–strain plot.

#### 2.2.3. Functional Characterization

##### In Vitro Hydrolytic and Enzymatic Biodegradation

The degradation of freshly prepared chitosan hydrogels was evaluated by immersing hydrogels in PBS solution in the presence and in the absence of lysozyme (1 mg/mL in PBS solution) at pH 7.4 and 37 °C. The quantification of the in vitro biodegradation (Equation (6)) was determined by weighing the mass loss at different time intervals:

Mass loss (%) = (100 − (W_0_ − W_t_)/W_0_) × 100 (6)

where W_0_ is the weight of the swollen hydrogel at initial time and W_t_ at t_∞_. Three samples were evaluated for each data point.

##### In Vitro Cytotoxicity Assay

Biocompatibility of the hydrogels was estimated using a live–dead assay. A total of 24 h before the assay, mouse embryonic fibroblasts (MEFs) were seeded in a 24-well plate at a density of 2.105 cells per well and cultured under standard conditions (37 °C and 5% CO_2_) in complete medium (DMEM containing 10% fetal bovine serum and 1% penicillin). Chitosan hydrogels (5–10 mg) formed with NaOH solutions of increasing molarity were washed thoroughly with D-PBS, dried and sterilized under UV light for 1 h before being added to the wells containing MEFs. After 24 h, hydrogels were discarded, and cell cultures were washed with PBS and stained with Calcein-AM (2 µM), ethidium homodimer (EthD-1, 4 µM) and NucBlue (Thermo Fisher (Waltham, MA, USA, R37605, 2 drops/well). Fluorescence images of the blue (NucBlue, cell nuclei), green (calcein, cytoplasm of live cells) and red (EthD-1, nuclei of dead cells) nuclei acquired using a Leica DMi8 fluorescence microscope, and the collected data were quantified using Fiji software [25]. Cell viability was calculated as the ratio between red-stained nuclei and the total number of cells (blue-stained nuclei) in at least five images for three independent samples.

##### Actuator Bending Response

Actuator bending response of prepared chitosan-based hydrogels was evaluated under electrical stimuli. For this, printed chitosan hydrogel strands were placed in a 0.1 M NaCl electrolytic solution between two platinum electrodes separated with a gap distance of 2 cm. Subsequently, a potential difference of 15 V was applied with a Hewlett-Packard E3615A DC power supply (0–20 V), and images were acquired using a digital camera.

## 3. Results

### 3.1. Synthesis and Swelling of Chitosan-Based Hydrogels 

**Figure 1:**
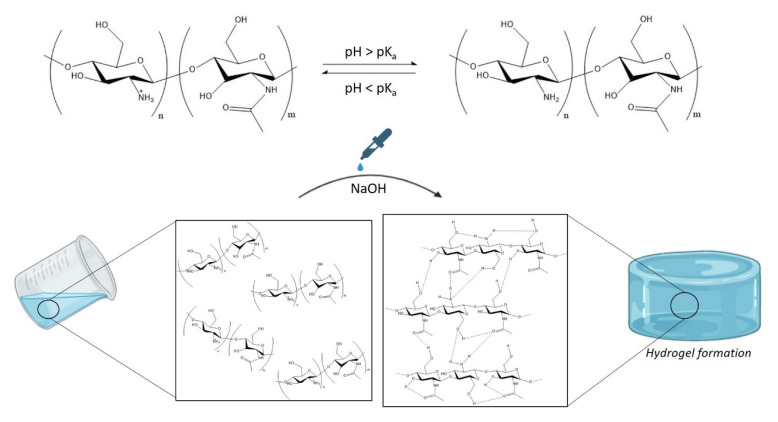
Schematic representation of chitosan-based hydrogel formation.

Chitosan is not soluble in water or physiological media; however, a chitosan solution can be obtained in acid media when the solvent’s pH is below its pKa value (pKa_chitosan_~6.5) [26]. In acidic aqueous media, the protonation of the primary amine groups present in the polysaccharide backbone takes place, leading to positively charged polymer chains and favouring electrostatic repulsion between chitosan chains. However, when a strong base (NaOH) is added to the media, chitosan remains stable in the solution until the pH becomes higher than its pKa value, which leads to the formation of hydrated gel-like precipitate [27]. This gelling mechanism is driven by the neutralization of primary amine groups, which promotes extensive hydrophobic interactions and hydrogen bonds among different moieties, such as amines, hydroxides and carbonyl groups present in the polysaccharide backbone (Figure 1). Thus, chitosan hydrogels can be formed by controlling chitosan concentration as well as the pH of the precipitating solution [28].

**Figure 2:**
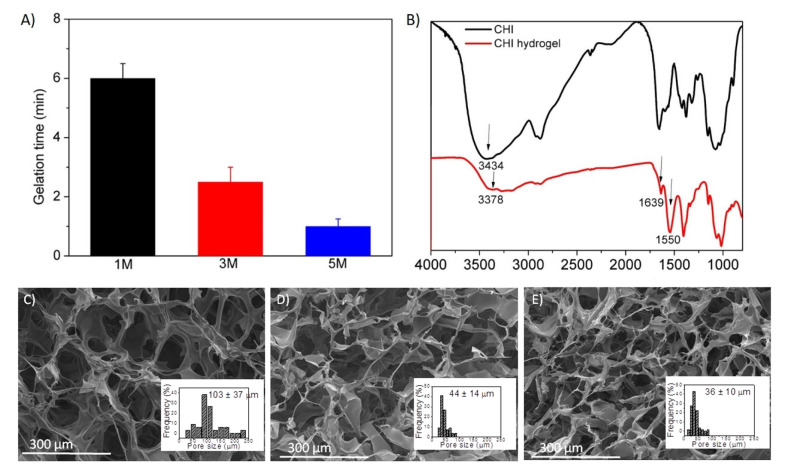
(**A**) Coagulation time of hydrogels prepared with different ionic strength, (**B**) FTIR spectra of pristine chitosan (black) and chitosan-based precipitate like gel (red) and SEM micrographs of hydrogels prepared by coagulation with NaOH (**C**) 1 M, (**D**) 3 M and (**E**) 5 M.

The gelation times (t_gel_)of prepared hydrogels were evaluated by the inverted tube test (Figure 2A), which lead to the discovery that, as the concentration of NaOH increases, lower times are required for the formation of the hydrated gel-like precipitate, which was 5 times lower in the case of NaOH 5 M than in the case of NaOH 1 M. 

FTIR spectra of both pristine chitosan and hydrated gel-like precipitate that are compared in Figure 2B corroborated the above-described gelling mechanism. On the one hand, a slight shift to lower frequencies was observed in the band at 3434 cm^−1^ of the gelled chitosan that was assigned to O−H stretching vibrations, which points out the reinforcement of hydrogen bonding. On the other hand, an increased intensity in the bands at 1639 and 1550 cm^−1^ (assigned to N−H deformation vibrations), attributed to hydrophobic interactions and molecular chain entanglement, can be also appreciated in chitosan hydrogels [1].

Further, hydrogel formation was also evaluated by SEM micrographs, shown in Figure 2C–E (for NaOH 1, 3 and 5 M, respectively), in which it is confirmed that the interactions established between polysaccharide chains lead to interconnected porous three-dimensional structures. These results are in agreement with the literature on this type of physical hydrogels [29,30].

In addition, a slight dependence of the pore size of the hydrogels and the concentration of the NaOH solution can be observed in Figure 2C–E. Although a low concentration (1 M) of a strong base such as NaOH caused the precipitation of chitosan hydrogels, it seems to have led to a decrease in hydrogen bonds or hydrophobic interactions within the polymer, with respect to higher concentrations of NaOH (3 M and 5 M), resulting in more open networks with remarkable larger pore dimensions (~103 μm vs. ~40 µm).

**Figure 3:**
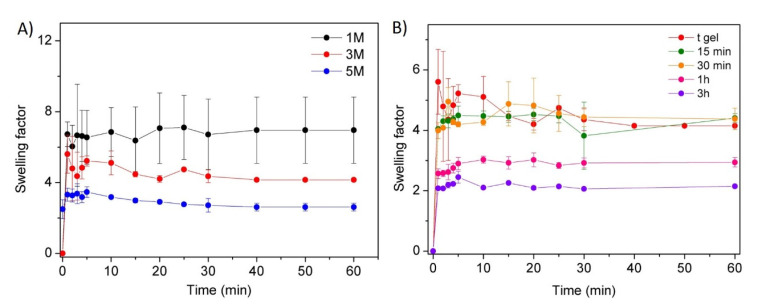
Swelling response of CHI/NaOH hydrogels. Influence of (**A**) NaOH ionic strength and (**B**) crosslinking time for CHI/NaOH 3 M hydrogels.

Furthermore, the influence of the NaOH solution concentration and coagulation time on the swelling behaviour of the hydrogels was also studied, and collected data are shown in Figure 3A,B.

Figure 3A shows a clear dependence of hydrogel swelling on the NaOH concentration. Certainly, the high pH values of the medium (NaOH 3 or 5 M) promoted chitosan intramolecular–intermolecular interactions, resulting in a higher crosslinked network with smaller pore size, as shown in the SEM analyses, which limited water absorption and, therefore, the swelling capacity of the hydrogels. Indeed, significantly lower swelling factors were obtained for NaOH 3 M (swelling factor ~5) or NaOH 5 M (swelling factor ~3) in comparison with NaOH 1 M (swelling factor ~7). On the other hand, the influence of the coagulation time for a fixed concentration of sodium hydroxide (3 M) on the swelling capacity of the prepared materials was also studied (Figure 3B). Higher coagulation times favoured the establishment of hydrogen bonds and/or hydrophobic interactions, increasing crosslinking density and, consequently, leading to networks that are more compact with a lower swelling ability. This influence reveals that despite the fact that gel formation occurs in situ (t_gel_), the establishment of interactions continues over time (t_∞_~3 h).

### 3.2. Mechanical and Rheological Properties 

**Figure 4:**
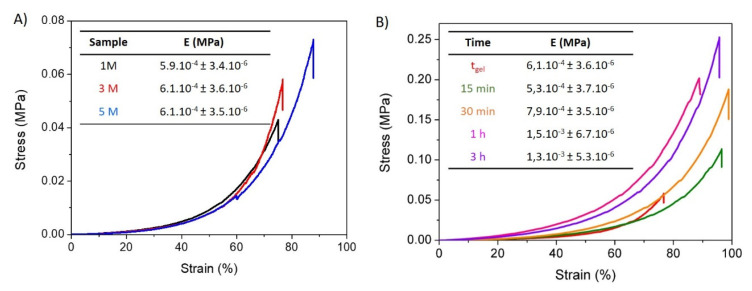
Mechanical compression stress–strain tests. Influence of (**A**) ionic strength of the gelling medium and (**B**) of the gelling time for CHI/NaOH 3 M hydrogels.

The unconfined compression tests of freshly prepared chitosan-based hydrated gel-like precipitates are shown in Figure 4A,B. These data reveal the influence of the concentration of NaOH solution and the exposure time to this solution on the mechanical stability of the studied materials. In both figures the typical non-linear mechanical response for this type of physical hydrogels when they are subjected to compression tests can be observed. However, chitosan-based hydrogels present a significantly different ability to deform depending on the concentration of the medium and the exposure time.

As is presented in Figure 4A, all hydrogels displayed a similar compression modulus (~6.1.10^−4^ MPa); however, an increasing stress was required to reach their maximum deformation as NaOH solution concentration increased. In this sense, the gel formed by 5 M NaOH showed the largest resistance to deformation and required the highest stress to reach its maximum deformation (89%). This may be a consequence of the higher interactivity between polymer chains derived from a more effective coagulation, leading to higher crosslinking density. These results are in good agreement with morphological and swelling data. In contrast, when the concentration of NaOH decreased to 1 M and 3 M, lower deformations at breakage and lower stress were needed for maximum deformation. As previously shown, the use of a lower ionic strength results in less interconnected three-dimensional networks, leading to softer hydrogels.

The influence of the crosslinking time on the mechanical stability was also evaluated (Figure 4B) for hydrogels formed by the addition of NaOH 3 M. Higher complexation times led to a higher force needed for breakage, and higher Young moduli were measured for the hydrogels with the longest crosslinking times. These effects can be ascribed to the higher number of interactions established between chitosan chains, resulting in higher crosslinking density, which is in agreement with above-presented results.

**Figure 5:**
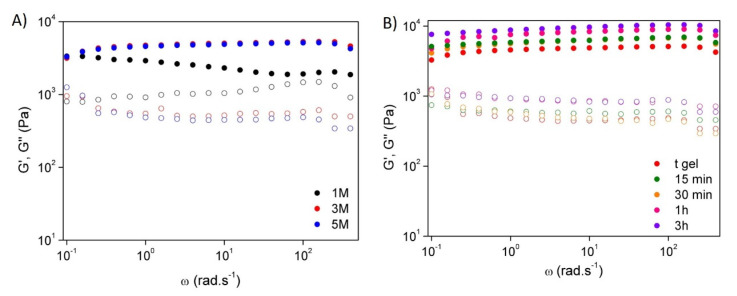
Rheology measurements. Storage modulus (G’) and loss modulus (G″) of the CHI-based hydrogels as a function of frequency: influence of the (**A**) ionic strength and (**B**) crosslinking time for hydrogels prepared with NaOH 3 M.

The rheological behaviour of the hydrogels was evaluated by frequency sweep tests at room temperature in order to study the effect of sodium hydroxide solution concentration (Figure 5A) and coagulation time (Figure 5B). It was noticeable that all samples behaved like a hydrogel once the storage modulus (G’) was higher than the loss modulus (G’’) in all frequency ranges. Furthermore, as the concentration of NaOH increased, both G’ and G’’ increased, leading to an improvement of the rheological properties, especially for those hydrogels prepared with NaOH 3 M and 5 M, which presented similar behaviour. Additionally, hydrogels formed by the addition of NaOH 3 M showed a slight improvement in their rheological properties with increasing coagulation time. This behaviour, as was also revealed in the mechanical properties, can be ascribed to the higher number of interactions established between chitosan chains over time, which leads to the formation of elastic auto-stable materials, i.e., strong hydrogels [31].

### 3.3. In Vitro Hydrolytic and Enzymatic Biodegradation

**Figure 6:**
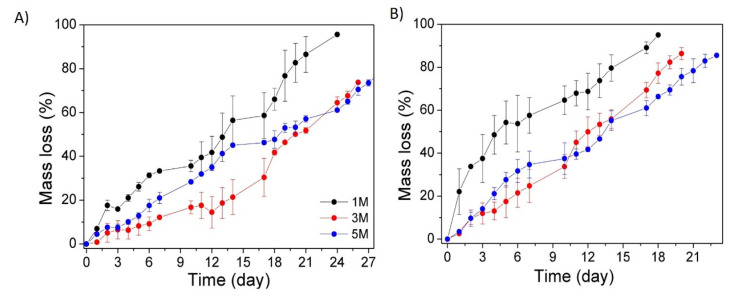
Degradation kinetics of the hydrogels prepared with different NaOH concentrations and different degradation media. (**A**) Hydrolytic media and (**B**) enzymatic media (lysozyme, 1 mg/mL).

The evaluation of the degradation kinetics of the hydrogels is an essential factor since it determines their stability and, as a consequence, their applicability. Thus, the degradation profiles of chitosan-based hydrogels prepared with different NaOH concentrations (1, 3 and 5 M) were studied in vitro at 37 °C and pH 7, as presented in Figure 6. The study was carried out in the presence and in the absence of lysozyme. This enzyme was specifically used to catalyze the biodegradation of chitosan since it favours the hydrolysis of β-(1,4) glycosidic linkages.

In general, it can be observed that hydrogels with a remarkably higher swelling ability, such as those coagulated with 1 M NaOH solution, experienced faster and larger mass loss regardless of the presence of the enzyme as a consequence of their more open networks. In addition, the presence of the enzyme accelerated (~30%) the mass loss of the hydrogels.

It can also be observed in Figure 6 that no major differences were observed between the different hydrogels prepared with variable ionic strengths, as in all cases degradation was achieved, which was almost complete after 20 days, in agreement with the literature for similar hydrogels [16,32].

### 3.4. In Vitro Cytotoxicity Assay 

**Figure 7:**
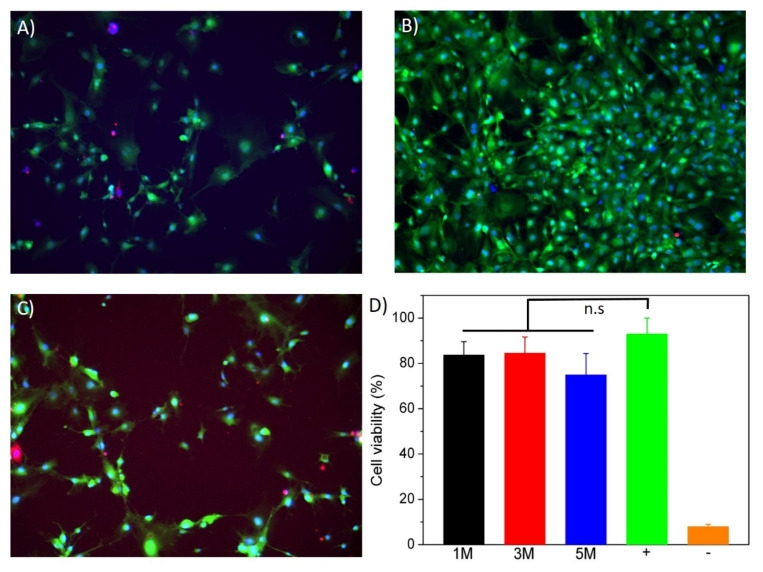
(**A***–***C**) Fluorescent images corresponding to CHI-based hydrogels coagulated with (**A**) NaOH 1 M, (**B**) NaOH 3 M and (**C**) NaOH 5 M. (**D**) Normalized cell viability results. In the positive control conditions, cell membranes were permeabilized by incubation in ethanol. Cells in the negative control conditions were cultured in absence of hydrogels. One-way ANOVA test with Tukey’s multiple comparison test was used for the statistical analysis (*p* < 0.05). (n.s) No significant differences.

To test the biocompatibility of chitosan gels obtained by NaOH precipitation, embryonic mouse fibroblasts (MEFs) were cultured in the presence of small fragments of hydrogels (5–10 mg) prepared with increasing molarities of NaOH (1, 3 and 5 M). After 24 h, cell viability was assessed using a fluorescent staining of the cytoplasm of live cells (calcein, green channel), the nuclei of all cells (NucBlue, blue channel) and the nuclei of dead cells (EthD-1, red channel) (Figure 7A–C). Cell viability was calculated as the ratio between red-stained and blue-stained nuclei (Figure 7D) and did not reveal any statistical differences between the control cells cultured in absence of hydrogels and the experimental conditions. This result exposes the excellent biocompatibility of the chitosan scaffolds independently of the molarity of the NaOH solution used to produce them.

### 3.5. Hydrogel Printability

**Figure 8:**
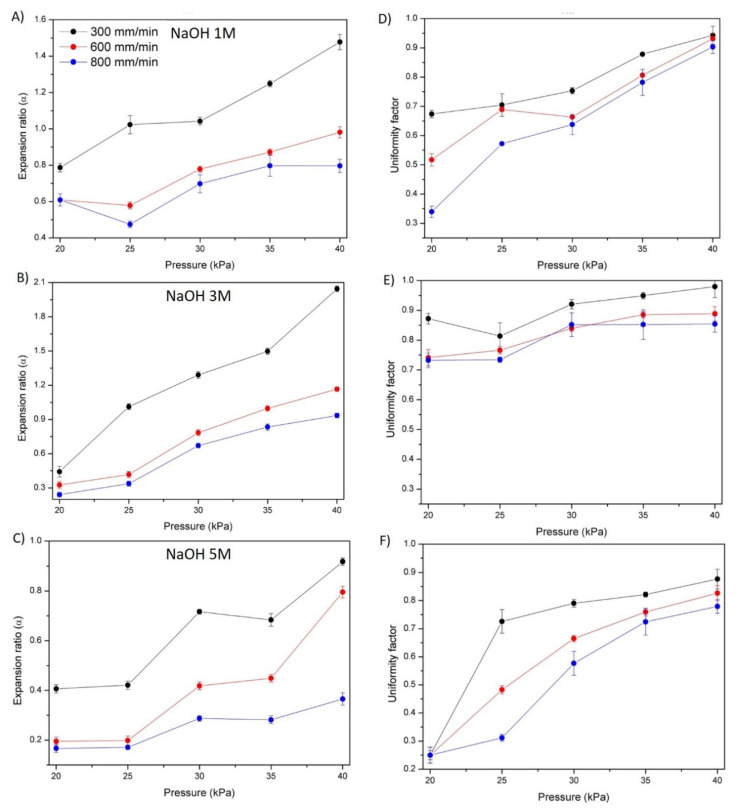

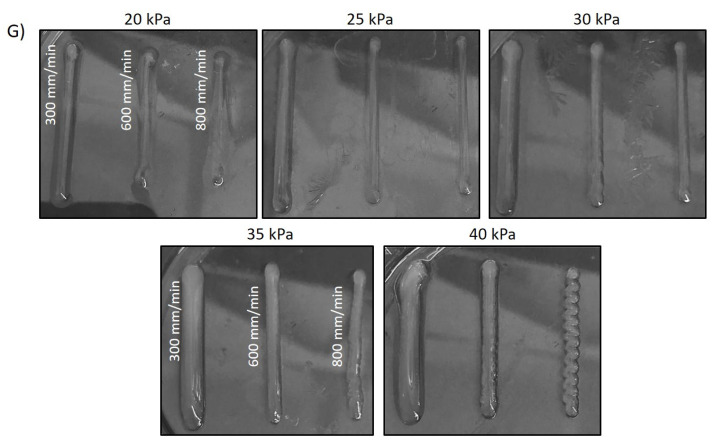
Chitosan hydrogel based on 1.5% *w*/*w*. solution with different NaOH concentrations. (**A**–**C**) expansion factors and (**D**–**F**) uniformity factor. (**G**) Digital photographs of CHI hydrogel coagulated with 3 M at different speeds and pressures with a nozzle of 25 G.

3D bioprinting technology has become a promising technique to fabricate scaffolds with high accuracy and precision, allowing the development of precise biomimetic 3D structures. Thus, hydrogels that can be used as bioinks for 3D printing technique represent a critical component in the preparation of hydrogel-based scaffolds for biomedical applications. The printability of easily gelled chitosan hydrogels was evaluated (Figure 8A–F) in order to optimize the printing parameters, such as speed and pressure, as well as to analyze the influence of the coagulating solution (NaOH 1, 3 and 5 M).

**Figure 9:**
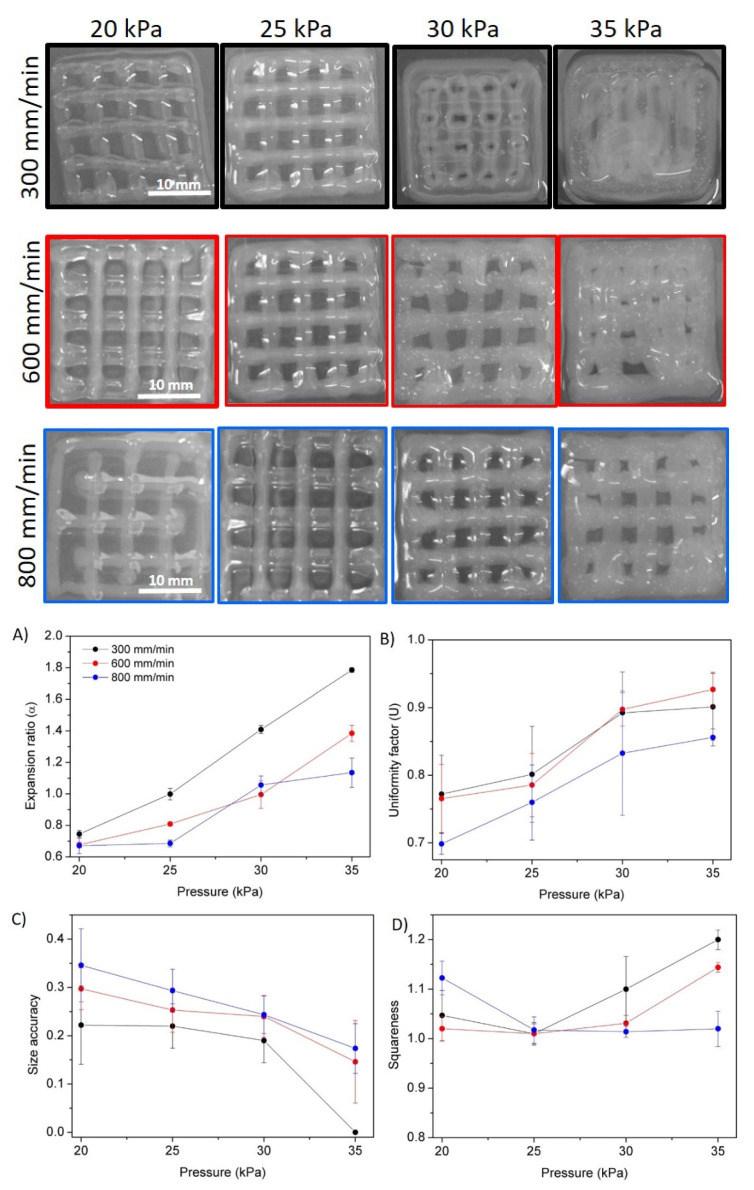
Digital images of printed square scaffolds of CHI/NaOH 3M hydrogel at different pressures and extrusion speeds with a 25 G nozzle. (**A**–**D**) Evaluation of the quality of printed structures: (**A**) expansion ratio, (**B**) uniformity factor, (**C**) size accuracy and (**D**) squareness.

In all cases and regardless the ionic strength of the NaOH coagulating solution it was observed that a decrease in printing speeds and an increase in extrusion pressure led to higher values of both the expansion and uniformity factors. Indeed, intermediate pressures (30 kPa) only led to the optimal expansion rate for gels prepared with 1 and 3 M gelling solutions. It was also observed that at slower speeds the material had more time to spread over the surface, leading to extremely high expansion ratio values for high pressures. However, one of the main advantages of printing at low speeds, as observed in Figure 8, is that the uniformity factors were strongly improved, reaching values close to 1 for high pressures (40 kPa). These results are in agreement with recent published results on related materials [33]. Thus, there is a compromise between expansion and uniformity when intermediate pressures are employed, and higher accuracy is promoted by intermediate pressures and slow speed rates. With respect to ionic strength, it was observed that as the NaOH solution concentration increased, ink spread decreased as its precipitation occurred more rapidly, as 3 M is the optimum concentration, leading to expansion ratio and uniformity factor values closer to 1 for intermediate pressures. The 5 M concentration promoted fast gelling that leds to a decrease in the printing accuracy. Thus, an intermediate ionic strength (3 M NaOH) was selected as the optimal ionic strength for printing pure chitosan (1.5% *w*/*w*)-based inks. The digital images of the strands printed in the different conditions with 3 M NaOH and a 25 G nozzle are shown in Figure 8G. Accordingly, under these conditions chitosan scaffolds with square geometry were printed (Figure 9).

Figure 9 shows that the appearance and definition of the square scaffolds varied significantly depending on the pressure and the speed at which they were printed. The combination of intermediate pressures with slow printing speeds favoured the printing of scaffolds with improved resolution and precision since less material was deposited at a time in the printing surface, which in turn led to a reduced pooling or collapse of the strands and vertices of the printed scaffold. Intermediate pressure (25 kPa) was found to promote optimum squareness, while no significant changes for variable speed rates were measured. However, it was observed that size accuracy differs significantly between the different printed scaffolds. At higher pressures and lower speeds, due to a larger flow of material and more time to spread, shape fidelity decreased sharply. This deterioration is reflected in the fact that the cavities of the printed pores did not maintain the theoretical area and instead began to fill with the bioink, which decreased their size and even filled the entire cavity. Accordingly, conditions of 30 kPa and 600 mm/min were shown to be the optimal printing conditions, that is, those that best promote expansion ratio, size accuracy, uniformity factor and squareness values closer to 1, leading to the highest quality of the printed chitosan scaffolds.

### 3.6. Bending Actuator

Hydrogels based on polyelectrolytes constitute interesting materials in the area of electroactive hydrogels due to their ability to locally swell and shrink in the opposite side under electric field, promoting their electromechanical bending response. Under an electric field, materials are capable of transforming electrical energy into mechanical energy due to the migration of ions in solutions, which leads to the swelling of one side of the hydrogel and the contraction of the opposite side [34,35]. Chitosan is a weak base with numerous ionizable primary amine groups along its structure that are able to induce this reversible electrical response.

**Figure 10:**
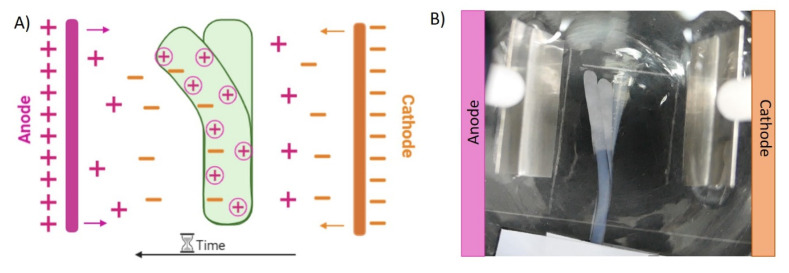
(**A**) Illustrative representation of the migration of ions through the hydrogel and electrolytic solution. (**B**) Bending behaviour of the CHI/NaOH 3 M strand (15 v).

Due to this, the electrically induced bending of the chitosan hydrogels was observed (Video S1). As is represented in Figure 10 printed chitosan strands bent towards the anode when a potential difference of 15 V was applied through two platinum electrodes. It is important to highlight that this bending behaviour is reversible; when the polarity of the electric field was inverted, chitosan strands bente back to the anode.

There are different theories to explain the behaviour of electroactive hydrogels when an electric field is applied. Flory’s theory of osmotic pressure is typically used for the explanation of polyelectrolyte-based hydrogels’ bending behaviour [36]. In this case, when an electric field is applied through the material, the free ions present in solution (Na^+^ and Cl, and in this case possibly OH^−^ residues) move toward their counter electrodes, leading to an osmotic pressure difference (∆π) along the direction of the potential difference inside and outside the hydrogel, due to the differences in concentration of these ions [21]. Consequently, in polycationic hydrogels (∆π < 0) the hydrogel swells on the cathode side while it shrinks in the anode, therefore bending to the anode side. In addition, the application of an electric field produces the electrolysis of the NaCl solution of the electrolytic media. In the negative pole (cathode), hydroxyl (OH^−^) groups are created, and hydrogen gas is produced (2H_2_O + 2e^−^→ 2H_2_(g) + OH^−^). On the other hand, in the positive pole (anode), chlorine gas is formed due to the consumption of chloride ions (2Cl^−^ → Cl_2_(g) + 2e^−^). In this way, the produced hydroxyl groups move towards the anode while the free sodium cations that are present in the electrolytic solutions travel up to the cathode [34]. Within chitosan-printed strands, ionized amine groups (positively charged) are balanced by the negatively charged ions (OH^−^) that are moving from the cathode to the anode and, at the same time, they are repelled from ions in the cathode (Na^+^) (Figure 10A). This fact makes the hydrogel swell on the side that is closest to the cathode and shrink on the anode side, thus bending toward the positive electrode [21,34]. Taking this electric response into consideration, the hydrogels showed a bending response suitable for the development of bioinspired soft actuators for soft robotics, allowing the further development of dynamic scaffold-based microenvironments for cell regeneration in biomedical applications [37].

## 4. Conclusions

Chitosan-based hydrogels were successfully prepared by coagulation using a strong base, such as NaOH, with different ionic strengths. The CHI/NaOH gel-like precipitates exhibited ionic strength dependence since their properties, such as morphology, swelling, mechanical stability and rheology, varied significantly when the ionic strength was changed. The printing process was optimized as a function of the disposal pressure, speed rate and gelling solution concentration. An intermediate concentration (3 M), pressure (30 KPa) and speed (600 mm/min) were shown to lead to the highest printing accuracy of square scaffolds. The study of the biodegradation process in the presence and in the absence of the enzyme showed no influence on the composition of the hydrogel; however, the presence of lysozyme in the degradation media accelerated the hydrolysis of O-glucosidic bonds present in chitosan backbone. In addition, it is important to highlight the ability of the hydrogel to deflect toward the anode when an electric field of 15 V is applied. Finally, the cytotoxicity tests showed the high biocompatibility of the gels, especially those coagulated with NaOH 1 and 3 M, which reveals their potential for the development of three-dimensional biomimetic scaffolds and as robotic actuators in medical applications.

## Data Availability

The data presented in this study are available on request from the corresponding author.

## Supplementary Materials

The following supporting information can be downloaded at: https://www.mdpi.com/article/10.3390/polym14030650/s1. Video S1: CHI/NaOH 3M hydrogel strand electrically induced bending.
